# St. Thomas's Hospital

**Published:** 1831-05

**Authors:** 


### ST. THOMAS'S HOSPITAL.

*St. Vitus's Dance.* [From Dr. Elliotson's Clinical Lecture.]

The other case, gentlemen, to which I beg leave particularly to direct your attention was that of St. Vitus's Dance. I last week stated that I had presented a patient with it cured, who had had the disease two years: that patient was a girl; this patient was a boy. The disease occurs much more frequently in girls than in boys, and the proportion of girls to boys who labour under it is very great. Dr. Heberden says, that in his experience one fourth only of the patients who had this disease were males and three fourths females. I made a calculation from my own experience in this disease during six years in the hospital, and I found the proportion about the same as had been observed by Dr. Heberden. Twenty-two females had had the disease, and but eight males. This is the opposite of what occurs in epilepsy: you will find the greater proportion of persons that have epilepsy are males.

This boy was fourteen years of age. You find that the greater number of individuals who have St. Vitus's dance are from six or seven up to sixteen or seventeen years of age. About the period of puberty, and a few years before, this disease is the most prevalent. He had it also about three years ago. You will find the recurrence of the disease very common, and I have frequently seen persons who have had it two, or even three times. I think I have observed the recurrences to take place more frequently in the spring than at any other time. I have formerly mentioned that there are in general no other symptoms in St. Vitus's dance except a little fatuity of look and mind. It is very common in this disease for children to be a little fatuitous, and look so; but as the disease is cured, both the look and the state of mind go away. Nothing, however, is more common than to find no other symptom than St. Vitus's dance. In epilepsy, you often have headach, giddiness, and a variety of similar symptoms; in many diseases of the nervous system you have constipation or tenderness of the abdomen, but here there is usually nothing of the kind. In the greater number of cases of St. Vitus's dance that I see, there is nothing but St. Vitus's dance, or at the utmost also some little pain in the head; but as to a disordered state of the digestive organs, I rarely find such a symptom in this disease. Children generally have their bowels regular; they have no diarrhœa; but of course sometimes children will have an affection of that description, or be costive, or have a slight inflammatory affection of the head, signs of fulness about the head; but none of these, I am quite satisfied, are essential to the disease. You may have these symptoms, but they are not essential to St. Vitus's dance.

With regard to the symptoms in this boy, he was in more or less constant motion; he could not walk straight, but twisted ftom one side to the other; his arms were flying about in all directions, and he made such faces when looking at you, and so wriggled his head, as to make one almost laugh: he was in perpetual motion. It is the character of the disease to have a catching of the fingers and other joints, twitches of the head, corrugation of the brow, and convulsions of all the muscles of the face, extensive flexions, and extensions and rotations of the limbs, perpetual motion, a rolling also of the eyes, and in walking you generally see one foot dragged after the other; such catchings of the tongue and of the muscles of the mouth and throat that articulation, deglutition, and mastication, are all impeded. This boy not only had twitches of the head, but could not speak; he did not speak for a length of time after he came to the hospital, with any distinctness. In severe cases, patients cannot lie in bed, and in still severer cases the convulsions continue during sleep, but in general they are suspended when the patient falls asleep.

The will has some little power over the motions. It appears that there is a strong inclination to these various motions, and the patient experiences a degree of pleasure in gratifying it. At any rate, for a moment, if you give patients a strong motive, they can arrest the motions, though only for a moment. If they be at all frightened, their irritability is increased, and the motions become much aggravated.

You will frequently observe that one side of the body is more affected than the other, as in so many nervous diseases; sometimes the disease is nearly confined to one side; and you will frequently find that, if you seize one arm and hold it tight, the other will be more agitated; and the same remark applies to the legs, and to an arm and leg.

The duration of the disease is very various, and there can be no doubt whatever but that in most cases, after a time, it will cease spontaneously, but, if left to pursue its course, will sometimes not terminate for a long period. The girl who was presented last week had had the disease two years; this boy, however, had only had it for a month.

In regard to the treatment, he took at his admission two drachms of subcarbonate of iron every six hours, and he never took any other medicine: but after he had been here some time, as the disease did not give way with great rapidity, I increased the doses to half an ounce; but had the two drachms relieved him as quickly as I could have wished, I should, of course, not have increased the dose: it was given him in twice its weight of treacle, and no aperient medicine at all was required. His diet was that of the house: there was no reason to lower his diet. There was no sign of fulness in the abdomen, no tenderness on pressure, no fulness of the head; no headach or giddiness except occasionally, such as children may accidentally have, but nothing at all to make low diet requisite. Under this treatment, with but one prescription, he got well. He was with us six weeks.

I have had now many dozens of cases of St. Vitus's dance, which have been all cured by this one remedy. There are other remedies which are exceedingly useful in the disease, and will cure it; but I think, compared with all others, this will cure the largest number of cases within a given time; I think so. I have not yet had a case in which I have failed with it; I mean a case which had existed only for a few months; was general, simple, and occurred in a very young person. The disease will sometimes be partial, and affect the head, or one particular limb, and continue for life in spite of every method you can adopt. It will sometimes be general in adults, and continue through life; but here it is generally united with some other nervous affection, perhaps with insanity or epilepsy. Except under these circumstances, however, I believe you will generally cure it with the subcarbonate of iron. I do not say universally, but I believe generally. There is, however, a great difference as to the time in which the remedy will cure. Usually I have cured it in from one month to two; but I have had cases where it was necessary to continue the remedy for twelve weeks before I made an impression on the disease, and then it yielded rapidly and went away. Some may be discouraged if they have to continue the remedy for many weeks; but it would be wrong to say it had failed unless it had been continued in some cases for three months; but these are extreme cases. However, if I found the case was not yielding to the remedy as quickly as I could wish, I would have recourse to other means at the same time, because the power of the medicine is now well established, and if other means are also well established, there is no reason why the patient should not have the full benefit of several. It is absurd to have recourse to more than one medicine if it answer the purpose; but you may wish to make the disease yield more readily, and, therefore, there can be no objection to resort at the same time to the cold bath, or electricity, or both; all of which have a certain power over the affection, and will cure a large number of cases. The sulphate of zinc has great power over the disease: it requires to be increased from a grain three or four times a day, to several grains; and you would be surprised at the number of grains which a person will take at last without nausea. I should certainly, unless the disease was giving way rapidly, increase it steadily as long as it produced no nausea.

With respect to the bowels, I paid no attention to them in this case, understanding that they were regularly open every day. Cases will get well under the use of purging, no doubt: but I have seen a great number of patients in the hospital, who had been briskly purged for a length of time before their admission, without getting at all better: indeed, some had grown worse, from being irritated and weakened; and the disease has at once given way under an opposite plan, tonic treatment. Old Meade, of this hospital, used to recommend iron in preference to any thing else in this affection. I presume, the subcarbonate of iron, if given in gruel, or mucilage, would constipate the bowels; but it is most probable that the treacle, in which I give it, counteracts the tendency. Treacle is an aperient substance, and if taken alone in large quantity would most probably produce diarrhœa; but I presume it counteracts the constipating effects of the iron, and the iron the laxative powers of the treacle. You might imagine, a priori, that the sulphate of zinc would constipate the bowels in this disease, it is so powerful an astringent, and it certainly is one of the most powerful astringents we have in the pharmacopœia; but it does not. I have frequently given from ten to twenty grains three times a-day without any constipated effect at all.

There is a form of the disease which I myself have never seen, but which is very extraordinary, and of which, gentlemen will recollect, I gave a full account in my general lectures on the theory and practice of medicine, where persons are seized with a violent impulse to regular motions. In common chorea the impulse is to irregular motions, but in the other form the movements are regular, so that the patients will have fits of dancing, and dance for hours, and it is said days, till they can keep up no longer, and down they go. Some have fits of running to a particular place; they will start from their home over the most dangerous places till they reach a particular place fixed in their mind, and then they drop down exhausted. Others are seized with fits of whirling round, pirouetting, and spinning and dancing, so that women, who have never been taught to dance have, under these fits, danced in the most graceful manner. You have the authority of a very respectable surgeon in the country for this, Mr. Kinderwood, who has furnished a case of this description recently in the Medico-Chirurgical Transactions. The disease, I believe, was first named from its appearing in this form. I need not say, that chorea signifies a dance; and the disease was first particularly noticed in some women in Germany, who were seized with fits of dancing, and went to the chapel of St. Vitus, near Ulm, and there danced till they were cured. You will find in that everlasting source of amusement, Burton's Anatomy of Melancholy, the following account:

"Chorus sancti Viti, or S. Vitus dance; the lascivious dance Paracelsus calls it, because they that are taken with it can do nothing but dance till they be dead, or cured. It is so called, for that the parties so troubled were wont to go to St. Vitus for help; and, after they had danced there awhile, they were certainly freed. It is strange to hear how long they will dance, and in what manner, over stools, forms, tables; even great-bellied women sometimes (and yet never hurt their children) will dance so long that they can stir neither hand nor foot, but seem to be quite dead. One in red clothes they cannot abide. Music, above all things, they love; and therefore magistrates in Germany will hire musicians to play to them, and some lusty sturdy companions to dance with them. This disease hath been very common in Germany, as appears by those relations of Sckenkius, and Paracelsus in his book of Madness, who brags how many several persons he hath cured of it. Felix Platerus (de Mentis Alienat. cap. 3) reports of a woman in Bazil whom he saw, that danced a whole month together."

If these cases had been described only in old works, they would have been treated with ridicule; but a great many things in old books are perfectly true, however strange they may appear; and it is often the explanations given of the occurrences that alone are preposterous. You will find a case mentioned by Dr. Watts, a most respectable physician at Glasgow, in the fifth volume of the Medico-Chirurgical Transactions. The patient, a woman, for most queer cases occur in women, had various motions at different times; she would roll over fifty or sixty times in a minute, and would be sometimes seized with a violent tetanic rigidity; and yet all the time she was perfectly conscious. You will find the case in the seventh volume of the same Transactions, related by Mr. Kinderwood, which also occurred in a female; she danced with grace, and was greatly delighted with music; and when a drum was beating, she danced up to it as close as possible, and yet, as I believe I mentioned, she never had learned to dance in her life. This woman also would take great pleasure in darting her finger into a hole in a screen, or darting it upwards against a given point in the ceiling. She sometimes would kneel down on one knee, with her hands behind her, and then suddenly spring up, and strike the ceiling with her hand, so that they were obliged to remove all the nails from the ceiling of the cottage, (she was a poor cottager,) lest her hands should be lacerated. It was observed, that in her there was a great fondness of music, exactly what Burton had previously mentioned, and it was noticed that a tune was to be heard from her mouth, if a person stood near her; and they therefore got a drum and beat it, and she was delighted beyond measure. It was by perverting her musical ideas that the disease was put a stop to. They found that if they beat out of tune or suddenly stopped, she instantly stopped, and in that way they broke the paroxysms. They found that if, instead of leaving off, they beat a continual roll, it had the same effect.

I presume these are affections of particular parts of the brain. I think there can be no doubt but that individual parts of the brain answer individual purposes, that every part has a particular function, and I think these cases cannot be explained but by the fact that certain parts alone are under a violent state of excitement in these affections. Great light has been thrown upon these cases, I think, by the experiments of Magendie, who, by cutting a certain part of the brain in an animal, caused it to have a fit of rolling. I recollect seeing him divide a certain part of the brain in a rabbit, when it instantly rolled round and round till it rolled to the edge of the table, and fell off upon the ground. When he divided another part, instantly the animal attempted progressive motion: it extended its paws and its head, and assumed the attitude of progression. In some cases of affections of the nervous system, it has been observed that persons have had a violent desire to run forwards, and in others to run backwards.

